# Mixed T Helper Cell Signatures In Chronic Rhinosinusitis with and without Polyps

**DOI:** 10.1371/journal.pone.0097581

**Published:** 2014-06-09

**Authors:** Lara Derycke, Stefanie Eyerich, Koen Van Crombruggen, Claudina Pérez-Novo, Gabriele Holtappels, Natalie Deruyck, Philippe Gevaert, Claus Bachert

**Affiliations:** 1 Upper Airway Research Laboratory (URL), Ghent University Hospital, Ghent, Belgium; 2 Division of ENT Diseases, KarolinskaInstitutet, Stockholm, Sweden; 3 ZAUM - Zentrum Allergie und Umwelt, München, Germany; Beijing Institiute of Otolaryngology, China

## Abstract

In chronic rhinosinusitis (CRS) different phenotypes have been reported based on cytokine profile and inflammatory cell patterns. The aim of this study was to characterize the intracytoplasmatic cytokines ofTcells infiltrating theinflamed sinonasal mucosa.

**Methods:**

Infiltrated T cells and tissue homogenates from sinonasal mucosal samples of 7 healthy subjects, 9 patients with CRS without nasal polyp (CRSsNP), 15 with CRS with nasal polyps (CRSwNP) and 5cystic fibrosis patients (CF-NP) were analyzed for cytokine expression using flow cytometry and multiplex analysis respectively. Intracytoplasmic cytokinesin T cells were analyzed after stimulation of nasal polyps with *Staphylococcus aureus* enterotoxin B for 24 hours.

**Results:**

The number of T cellsper total living cells was significantly higher in patients with CRSwNP vs. CRSsNP and controls. 85% of the CD4+ Tcells showed to be memory T cells. The effector T cells present in all tissues have apredominantTh1 phenotype. Only in CRSwNP, a significantfraction of T cellsproduced the Th2 cytokinesIL-4 and IL-5, while nasal polyps from CF patients were characterized by a higher CD4/CD8 T cell ratio and an increased number of Th17 cells. 24 h stimulation with SEB resulted in a significant induction of CD4+ T cells producing IL-10 (Tr1 cells).

**Conclusion:**

T cell cytokine patternsin healthy and inflamed sinonasal mucosa revealed that Th2 cells (IL-4 and IL-5 producing cells) are significantly increased in CRSwNP mucosal inflammation. Exposure to SEB stimulates Tr1 cellsthat may contribute to the Th2 bias in CRSwNP.

## Introduction

Last decenniumthe characterization of T cell subsets has accelerated the understanding of inflammatory and humoral immune responses in part by unveiling the enormous plasticity within these T cell subsets. The balance in T helper subsets as observed in healthy mucosa is disturbed in inflamed mucosa. CD4+ T cells are able to differentiate from naïve T cells into T helper (Th)1, Th2, Th9, Th17, Th22, or Tfollicular helper (Tfh) effector cell subset [1], [2] and this maturation process is largely dependent on antigen presenting cells such as dendritic cells, which mediate their effectvia the release of cytokines and cofactors. Similarly, CD8+ T cells can differentiate to cytotoxic T cell subsets: Tc1, Tc2, Tc17 cells. Moreover, memory T cells are able to switch from one to another type depending on the micro-environmental and mucosal factors [3].

T helper cell lineage differentiation is mediated by epigenetic processes. For example, naïve CD4 T cell differentiation into Th2 is accompanied by CpGdemethylation and histone modifications within the Th2 locus [4]. Each T cell lineage has distinct molecular, cellular and functional properties. Th2 cells were initially characterized as T cells expressing IL-4, IL-5 and IL-13. Each Th2 cytokine has a well-defined and relatively specific function. IL-4 is the factor driving IgE class switching and alternative macrophage activation, whereas IL-13 functions as an effector molecule that induces physiologic changes of the airways and IL-5 is the major eosinophil activating cytokine [5], [6]. Th1 cells fulfill diverse functions in the immune system by secretion of IFN-γ and cytotoxic effects on target, while Th17 and Th22 cells have a very important function in anti-microbial immunity at epithelial/mucosal barriers. Tfh cells regulate the development of antigen-specific B cell immunity [2].

Chronic rhinosinusitis (CRS) is a highly prevalent inflammation of the nose and the paranasal cavities. CRS can be subdivided in CRS without nasal polyps (CRSsNP) and CRS with nasal polyps (CRSwNP) based on clinical parameters, different cytokine pattern and distinct cellular profiles [7]. Current data on cytokines expression within sinonasal homogenates [8] suggest that maintenance of mucosal health and/or inflammation is not dependent of one T helper cell subset but rather the contribution of several subsets expressed at the same time. Recently different studies demonstrating the presence of IL-5, IL-17 and IFNγ, eosinophilic cationic protein (ECP), myeloperoxidase (MPO) and local IgE in homogenatesfrom CRSwNP tissue support the presence of different T cell subsets and consecutive variations in the inflammatory patterns [9], [10]. In CRSsNP however, no IL-5 could be detected on protein level by multiplex analysis, but only IFNγ protein could be observed, pointing to Th1 cells as orchestrators.

The aim of this study was to investigate the relative presence of CD4 and CD8 T cells in sinonasal mucosa of healthy controls, CRSsNP and CRSwNP patients and to study intracytoplasmatic expression of cytokines by these T cell populations. This study is describing the different T helper cell populations in healthy and inflamed nasal mucosa by performing polychromatic flow cytometry.

## Materials and Methods

### Patients

Patients were recruited at the department of Otorhinolaryngology of the Ghent University Hospital, Belgium. Inferior turbinate samples from patients without sinus disease undergoing septoplasty or rhinoseptoplasty were collected as controls (controls n = 7). Samples from patients suffering from chronic rhinosinusitis (CRSsNP n = 9, CRSwNP n = 15 and cystic fibrosis (CF)-NP n = 5) were obtained during functional endoscopic sinus surgery procedures. The study for collecting human tissue samples was approved by the ethical committee of the University of Ghent, Belgium. The number appointed to the study: B67020072535. A written informed consent was obtained by all patients. The diagnosis of sinus disease was based on history, clinical examination, nasal endoscopy and computed tomography of the paranasal cavities according to the current European EPOS guidelines [11]. More detailed information can be found in this article’s Online Repository (file S1).

### Cytokine Measurements in Tissue Homogenates

Cytokine measurements were performed on tissue homogenates as previously described [12] and were assayed by using the LuminexxMAP suspension array technology in a Bio-Plex 200 system (BioRad, MI, USA). For this, kits for IL-5, IL-17 and IL-1β were purchased from R&D Systems (Minneapolis, USA). Concentrations of IFNγ were determined with commercially available ELISA kit (R&D Systems). Total IgE, ECP and specific IgE to staphylococcal enterotoxins (SE-IgE) were measured by the UNICAP system according to manufactureŕs guidelines (Thermo Fisher Scientific-Phadia, Sweden).

### Preparation of Tissue Single Cell Suspension and Stimulation of T Cells

Fresh human nasal mucosa was processed as described as before [12]. To check T cell intracytoplasmatic cytokine expression, nasal mucosa single cells were resuspended in tissue culture medium (TCM, RPMI1640 with 10% FBS). T cells were activated with 50 ng/ml PMA (phorbol 12-myristate 13-acetate) (Sigma) and 1 µg/mlionomycin (Sigma) for 6 hours and incubated at 37°C and 5% CO_2,_ after 1 hour 3 µg/ml Brefeldin A (ebioscience) was added.

To test the effect of superantigens on T helper cell plasticity, nasal mucosa single cells were stimulated with 0,5 µg/ml *Staphylococcus aureus* enterotoxin B (Sigma) or TCM for 24 hours and the last 4 hoursPMA/Ionomycin/Brefeldin A was added.

### Characterization of T Cell Subtypes

Cells were harvested, centrifuged, washed and ready for intracellular cytokine staining (ICCS) to characterize and define the different T cell subsets. More detailed information can be found in this article’s Online Repository (file S1).

### Statistics

The data generated in this study were analyzed using the SPSS software version 19. More detailed information can be found in this article’s Online Repository (file S1).

## Results

### Clinical Characterization of Patients and Analysis of Key Cytokines in Sinonasal Tissue

Clinical characteristics of the different samples are described in table 1. Concentrations of IL-4, IL-5 and IgE were significantly higher in the CRSwNP group when compared to control tissue (p<0.01) as shown in table 2. Levels of IL-1β, IL-6, IL-8, IL-17, and IFNγ showed no significant difference between control, CRSsNP and CSRwNP groups, while in CF-NP, a significant increase of IL-1β and IL-17 vs. control and CRSwNP was observed.

**Table 1:** Patient description.

Clinical Features	Controln = 7	CRSsNPn = 9	CRSwNPn = 15	CF-NPn = 5
Age (years) Mean (SEM)	26,5 (3,13)	43,8 (4,44)	47 (4,06)	12,2 (2,33)
Female/Male, n/n	2/5	1/8	4/11	3/2
Allergy, n (%)	2 (28,5)	2 (22,2)	8 (53,3)	1 (20)
Asthma, n (%)	0	0	6 (40)	0

**Table 2:** Cytokine data on tissue homogenates.

Mediators	Control	CRSsNP	CRSwNP	CF-NP
IL-1β (pg/ml)	40,8 (14,7–133)	53,6 (30,4–323,7)	58,09 (9,7–1742)	532,7 (184–1299)*∇◊
IL-4 (pg/ml)	9,9 (7,6–23,4)	12,2 (7,59–41,47)	32,9 (7,6–89,4)*	14,3 (7,6–31,5)◊
IL-5 (pg/ml)	BDL	14,4 (6,5–267)*	175,3 (6,5–2126,7)*∇	6,5 (6,5–16,83)
IL-17 (pg/ml)	12,5 (12,5–56,1)	12,5 (12,5–379,28)	12,5 (12,5–130,4)	209,8 (12,5–519)*∇◊
IFNγ (pg/ml)	42,8 (41–286,9)	72,6 (41–941,5)	42,6 (41–273)	259,5 (183,6–286,3)◊
IgE (kU/L)	5,8 (1,9–18,7)	53,9 (1,93–222,6)*	445,5 (32,2–4533,1)*∇	56,8 (11,6–942,7)
ECP (µg/L)	247,3 (159,8–398,1)	1276 (507,3–2921,5)*	9856 (3663,3–15730)*∇	2310 (1705–4064,5)*◊

Data are expressed as median and interquartile range. P values signs after Mann-Whitney *U* test means as follow: *: p<0,05 in C versus CRSsNP, C versus CRSwNP or C versus CF-NP; ∇: p<0,05 in CRSsNP versus CRSwNP or CRSsNP versus CF-NP; ◊: p<0,05 in CRSwNP versus CF-NP.

### Phenotypical Characterization of Sinonasal Mucosa T Cells

**Figure 1:**
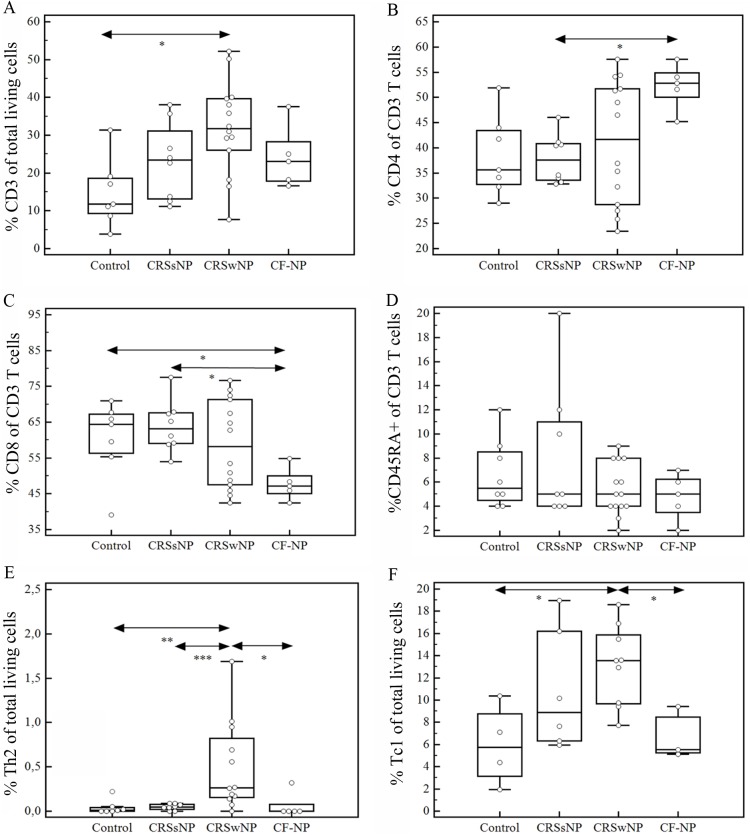
Flow cytometricanalyses of T cells in sinonasal mucosa. Data are expressed in Box-Whisker-plots presenting the results of CD3+ T cells (a), CD4+ T cells (b), CD8+ T cells (c) and the proportion CD45RA+ within the CD3+ T cell population (d). Box-Whisker-plot presenting the results of the percentage of Th2 cells in the different disease groups (e). Percentage of Tc1 cells (IFNγ producing CD8 T cells) in healthy and diseased nasal mucosa are presented as a Box-Whisker-plot (f). Significance (*p*) values after *Mann-Whitney U* test are represented by: * when p<0,05, ** when p<0,01 and *** when p<0,005.

T cellpopulations present in the nasal mucosa from healthy subjects, CRSsNP, CRSwNP and CF-NP patients were identified as CD3, CD4, CD8, and CD45RA T cellsby using multicolor immunofluorescence staining and flow cytometric analysis. The percentage (%) of CD3+ to total living cells (Figure 1a) was the highest in CRSwNP [31,7%, IQR: 24,05–39,8] and the lowest in healthy subjects [11,8%; IQR: 8,7–19,1], p = 0,011. The number of CD4+ and CD8+ T cells varied in function of the disease phenotype (Figure 1b, c). In CRSwNP [0,91; IQR: 0,52–1,19] and CF-NP [1,12; IQR: 0,94–1,28], the ratio CD4+ to CD8+ was significantly higher (p = 0,042) compared to CRSsNP [0,525, IQR: 0,48–0,68] mucosa, wheremore cytotoxic T cells were expressed. Moreover, 85% of the CD4 T cells were CD45RA negative which means that the T cells have almost all a memory/activated phenotype (Figure 1d); however there were no differences in the relative CD45RA expression between the different disease phenotypes.

### Cytokine Production by CD4+ T Lymphocytes

The intracellular cytokine pattern was examined by immunofluorescence staining and FACS analysis to delineate Th1, Th2, Th17, Th22 and Tfh cells. Under basal non-stimulated conditions littleto no cytokineswere produced (Figure S1a in file S1). However, after 4 hoursof incubation with PMA/Ionomycin in presence of Brefeldin A, different cytokines could be detected (Figure S1b in File S1) and differences dependent on the disease phenotype (control vs. CRSwNP) were observed. We observed no IL-9 positive T cells (Th9).

**Figure 2:**
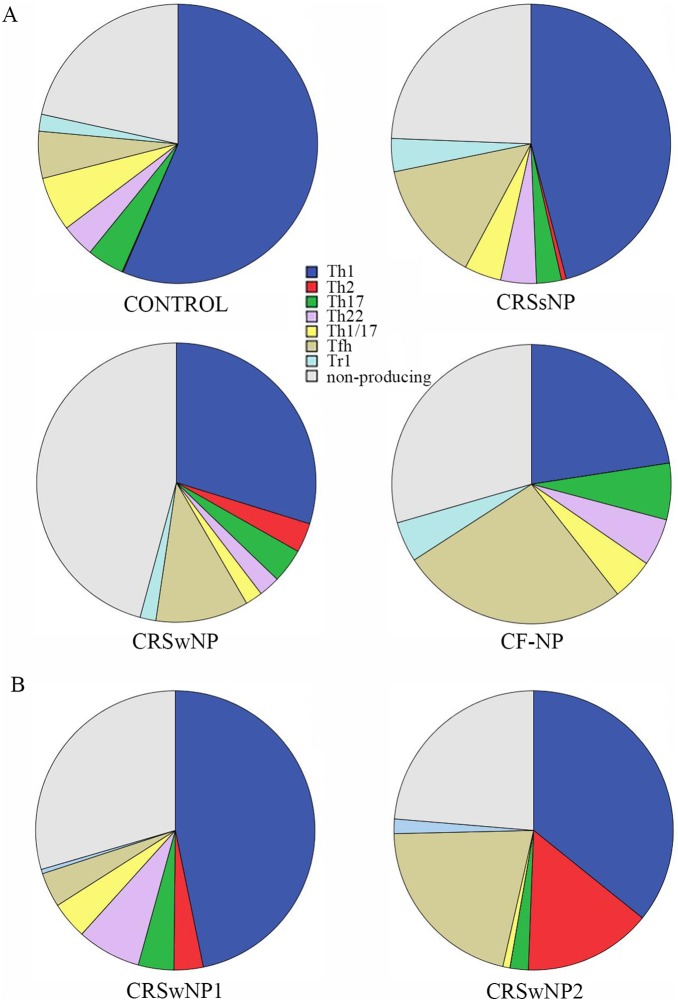
T cell subsets in healthy and diseased nasal mucosa. Different T cell subsets, namely Th1, Th2, Th17, Th22, Th1/Th17, T cell producing IL-10 or IL-21 and non-producing T cells were analyzed by flow cytometry. Median results for control, CRSsNP, CRSwNP and CF-NP are presented in pie charts. Pie charts presenting the variability in CRSwNP signatures. Scatter plots presenting the cytokine expression by CD4+ T cells without stimulus (a) and with stimulus PMA/Ionomycin (b).

The results obtained for the different T helper subsets in control and diseased mucosa are illustrated in Figure 2a. The variability between individual CRSwNP patients was remarkable and is demonstrated in Figure 2b using two individual patients.

Th2 (CD4+) cells producing IL5 and/or IL4 wereonly present in CRSwNP [0,41; IQR: 0,16–1,18], and negligible in nasal mucosa from controls [0,005; IQR: 0,000–0,05], CRSsNP [0,0497; IQR: 0,0092–0,0858] and CF-NP [0,04; IQR: 0,009–0,085] subjects as shown in (Figure 1e). After subgrouping the CRSwNP samples with respect to the asthmatic status, we observed that the asthmatic patients [0,67; IQR: 0,22–1,29] had significantly more Th2 cells compared to non-asthmatic patients [0,17; IQR: 0,04–0,64] (p<0,05).

When comparing CF-NP patientswith the other disease groups, a significant increase in Th17 and IL-21 [0,65; IQR: 0,33–0,68] producing T cells when observed compared to control subjects [0,20; IQR: 0,13–0,33]. A difference in Th17 cells was also observed to the CRSwNP [0,48; IQT: 0,28–0,72] group; however, it did not reach significance due to the limited number of CF-NP samples.

### Cytokine Production by CD8+ T Lymphocytes

Next to CD4+ T cells also the CD8+ T cells produced different cytokines. The major cytokine produced by 60 to 85% of CD8+ T cells was IFNγ. In CRSwNP [13,56, IQR: 9,58–16,20] the number IFNγ+-CD8 T cells (Tc1 cells) was significantly increasedwhen compared to control samples [5,72, IQR: 2,55–9,56] and CF-NP samples [5,54, IQR: 5,13–9,43] (Figure 1f). No differences in the number of these cells were found when patients were divided according their atopic or asthma status (data not shown). Moreover, +a small portion of IL-17 producing CD8+ T cells (Tc17 cells) was observed but without differences between the groups, whereas CD8+ expressing IL-4 and/or IL-5 were almost undetectable.

### Co-expression of the T Helper Cell Cytokines in Sinus Mucosa

**Figure 3:**
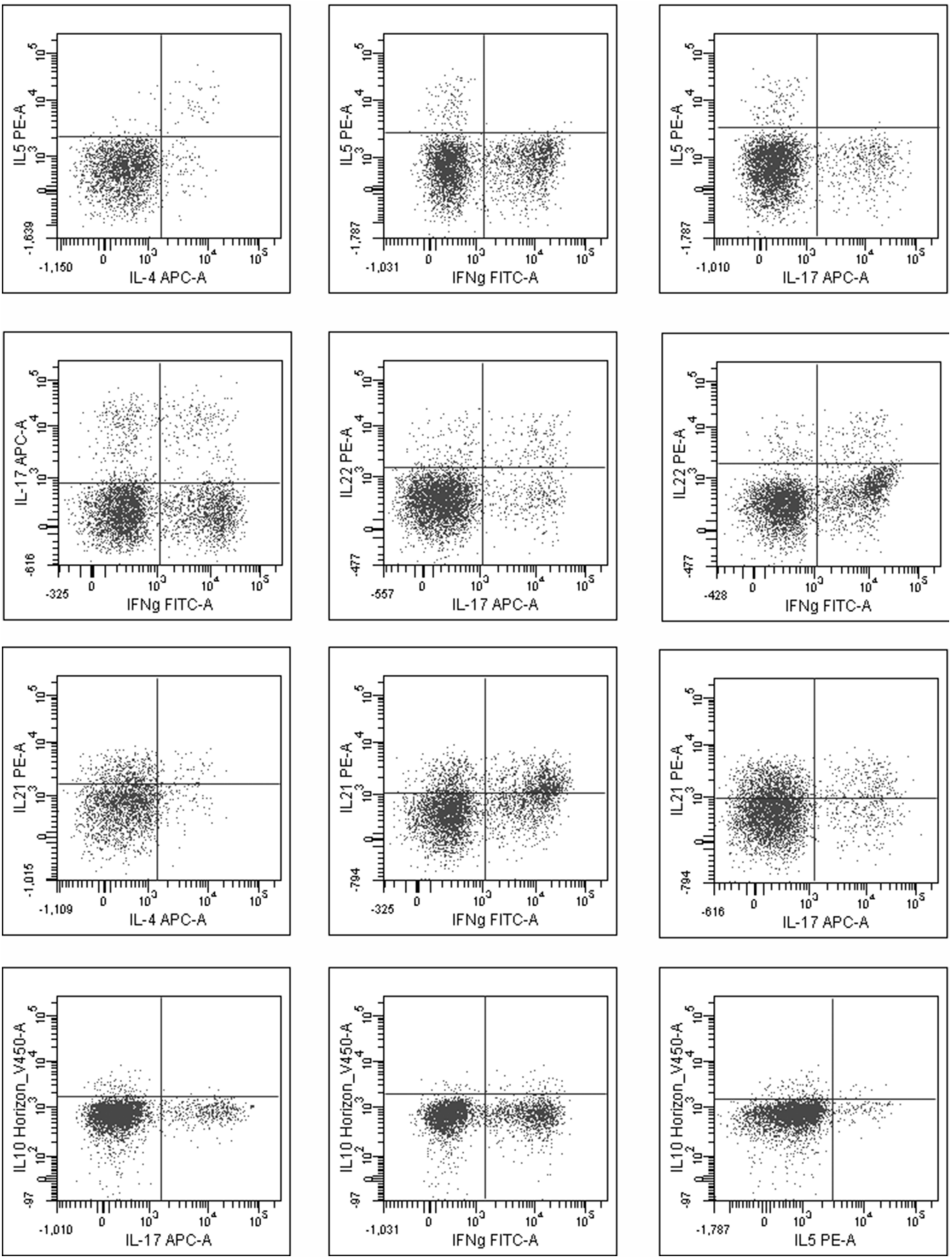
Cytokine co-expression in T cellsfrom nasal mucosa. Scatter plots presenting the co-expression of different cytokines in the same T cell. Co-expression of the Th2 cytokines: IL-5 with IL-4, IFNγ or IL-17 (first row). Co-expression of cytokines: IL-17 with IFNγ, IL-22 with IL-17 or IL-22 with IFNγ (second row). Co-expression of IL-21: with IL-4, IFNγ or IL-17 (third row). IL-10 producing T cells co-expression with IL-17, IFNγ or IL-5 (last row).

In the Th2 cell population, all IL-5+ CD4T-cells also produced IL-4, while a proportion of IL-4+ cells did not express IL-5. We also observed simultaneous production of IL-17/IFNγ, IL-17/IL-22/IFNγ, IL-17/IL21 and IFNγ/IL-21 (Figure 3) in CD4+ T cell population. Of interest, we could not detect any co-expression of Th2 cytokines (IL-4 or IL-5) with IL-17, IL-22 or IFNγ. A few CD4+ IL-4 secreting cells also produced IL-21 (Figure 3) suggesting the presence of functional T follicular cells in CRSwNP samples. We were able to observe more plasticity in the Th17 cell populationcompared to Th2 cells; however no differences between the disease subgroups were found.

### T Helper Cell and Global (Tissue Homogenate) Cytokine Expression

Cytokine results obtained by multiplex analysis on the tissue homogenates was compared with the data collected by flow cytometry. Concentrations for IL-5 protein significantly correlated with the number of Th2 cells observed by FACS (*r* = 0,856, p<0,05), indicating that the bulk of this Th2 cytokine is originatedmainly from T cells. The Th2 presence correlated also with an eosinophilic type of CRSwNP (ECP levels) (*r* = 0,587, p<0,05). The association between tissue IL-17 levels and Th17 cell numbers was significant, but less pronounced (*r* = 0,454, p<0,05) suggesting sources other than T-cells contribute to the production of this cytokine. For the Th1 cells we were not able to find a correlation because IFNγ may not be spontaneously released.

### Staphylococcus Enterotoxin B Changes T Cell Cytokine Pattern in CRSwNP

**Figure 4:**
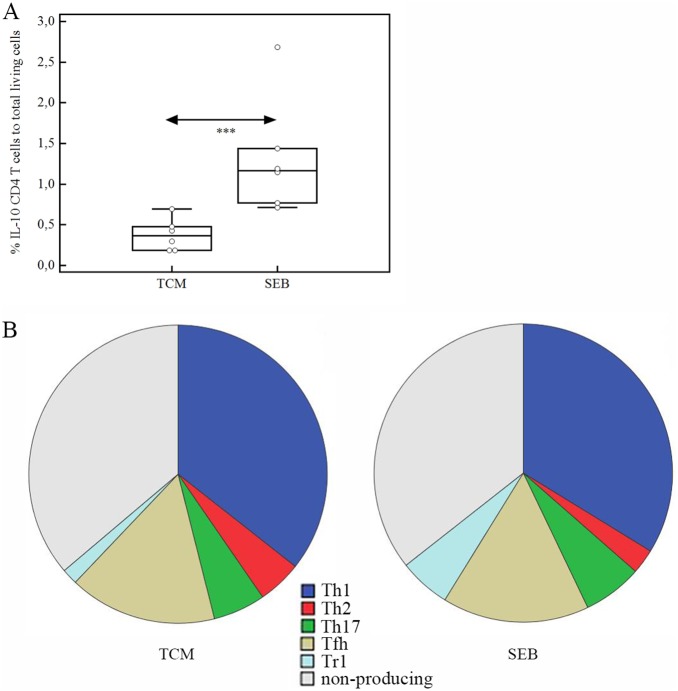
Influence of SEB on T cell cytokine pattern. Data are expressed in Box-Whisker-plots presenting the results of CRSwNP single suspension after 24 hours without and with 0,5 µg/ml SEB (a). Significance (*p*) values after *Mann-Whitney U* test are represented by: *** when p<0,005. Pie charts representing the median result of the different T cells subsets with and without SEB (b).

Former *ex vivo* SEB stimulation experiments with nasal tissue cubes showed a strong release of Th1, Th2 and Th17 cytokines [13]. Here, we questioned if the T cell profiles changed after 24 hours of SEB treatment compared tobaseline. A significant increase of IL-10 producing cells was observedafter SEB treatment [baseline 0,36; IQR: 0,19–0,53 vs. SEB 1,17; IQR: 0,76–1,75] (p = 0,004), as well as anon-significant increase of Th17 cells (Figure 4). Other T cell subsets did not change after SEB treatment.

## Discussion

In this study a detailedsubtyping of T cell populations present in diseased and healthy nasal mucosa was conducted by means of flow cytometry. Most T cells in the nasal mucosa were CD45RA negative and of an effector phenotype, which is in agreement with earlier data [14] and in opposite with the T cells circulating in the bloodthatpreferentially carry markers of the naïve T cell phenotype.

In healthy and diseased nasal mucosa a heterogenic population of T helper cells is present: Th1, Th17, Th22, and Tfh cells. The reason for this heterogeneity of effector T cells is mainly related to their protective function, enabling the best type of immune response according to the nature of the invading microorganism [1]. Our results show that the T cell profile of CRSsNP samples was similar to that of control samples, confirmingour earlier hypothesis that CRSsNP is a process of remodeling rather than an inflammatory disease [15]. Strikingly, by means of flow cytometry the Th2 cell subsets could only be detected in CRSwNP samples with the highest levels measured in asthmatic patients. This pattern is consistent with protein dataon homogenized tissue samplesusing multiplex cytokine analysis in a previous study of our lab [9] being corroborated by others [16]. Although IFNγ was the major intracytoplasmatic cytokine in T cells, it is almost undetectable in tissue homogenates; obviously, IFNγ is not spontaneously released on basal conditions while upon stimulation with SEB or PMA/Ionomycinhigh levels of IFNγ could be detected in cell free supernatants. Therefore, Th1 cells are present in CRSwNP mucosal tissue but have to be further triggered to release IFNγ. While IL-5 in comparison to IFNγ is only expressed by a small proportion of CD4+ T cells, it iseasily detectable in a subgroup of CRSwNP samples and associated with an increase in ECP.

Next to CD4+ T cells, an even larger population of T cells in the nasal mucosa belongs to the CD8 lineage, representing the major sourceof IFNγ. CD8+ cytotoxic T cells (Tc cells) are known to express high levels of Fas ligand which induces apoptosis in other cells. An important function of the IFNγ-producing CD8+ subset of T cells (Tc1 cells) is their ability to inhibit IgE responses independent from suppression of IL-4 producing cells but via the enhancement of Th1 responses [17]. A small percentage of CD8+ T cells expressed IL-17 and ina few nasal polyp samplesIL-5 could be detected. Effector CD8+ T cells are able to attenuate pulmonary inflammation by changing the polarization of T cells to a Th1 phenotype in a Th2 environment [18], however in asthmatic mice with a strong Th2 response to OVA; a concomitant CD8+ T cells response to viral infection may result in an increase of Tc2 cells, causing lung eosinophilia [17]. Furthermore, Rowe et al. [19] observed in children that IFN-γ production by CD8+ T cells may synergize with Th2 cytokines in driving atopy development. The exact functions of Tc1 and Tc2 cells have not been studied in CRS, but our data open perspectives for further research.

In all samples analyzed in this study, a certain co-expression/plasticity of T helpercells has been observed. Co-expression was especially determined in the Th17 cell population which produced not only IL-17, but also IFNγ and IL-22. Some authors annotate these T cells as alternative Th17 cells [20], these can easily change to a more Th1 phenotype or more Th17 phenotype depending on the external stimuli of the nasal mucosa. In contrast we could not observe Th2 cells producing IL-17 or IFNγ which is different from the data found in allergic asthma and atopic dermatitis patients [21].

Strikingly, although nasal polyps from patients with and without cystic fibrosis patients are morphological alike; they are initiated by different pathomechanisms and occur at different time pointsin life. A pathogen frequently associated with the development of Th2-biased CRSwNP is *Staphylococcus aureus,* while cystic fibrosis patients are frequently infected by *Pseudomonas aeruginosa* and are characterized as a Th17 disease [12]. Here weconfirm earlier dataof our group by reporting an increased presence of T cells secreting IL-17 and a large number ofIL-21 secreting T cells in nasal polyps from CF patients.

The data presented resulted from 7 to 15 samples analyzed in the control and CRSwNP groups, respectively, which is accepted in the literature for this kind of pilot biomarker investigation; however, the CF group with only 5 samples has to be considered small. On the basis of this pilot study, sample size calculations can be performed for further studies.

A high variability in Th2 cell cytokine expression was detected in the group of CRSwNP specimens: IL-4 expressing T cells represented 1 to 15% of the CD4+ T cells. Asthmatic patients had the highest number of Th2 cells, this observation also being linked to the expression of SE-IgE antibodies, confirming our earlier analysis based on protein data; those data also showed a great variety of cytokine expression in CRSwNP samples, and a highly increased risk of being asthmatic with the expression of IL-5 and SE-IgE [9].

Patou et al. [13] who performed *ex vivo* tissue stimulationexperiments, making use of nasal polyp tissue showed that SEB treatment induced a strong up-regulation of different cytokines including IL-2, IL-4, IL-5, IL-10, TNFα and IFNγ. However when treating single cell suspensionsof nasal polyp sampleswith SEBfor 24 hours, we demonstrated that SEB induces anup-regulation of T cells secreting IL-10, whereas Tcells expressing other cytokines were not increasing in numbers. IL-10 is an anti-inflammatory cytokine that can be expressed by different Th cell subsets, namely Th1, Th2, Th9, Tfh, nTreg, iTreg and Tr1 cells [22], [23], with theTr-like cells being the most potent source of IL-10. SEB stimulation induced FOXP3 positive T cells (data not shown), however, without anyco-expression of IL-10 in CD4+ T cells. Therefore, iTreg cells are here not a likely source of IL-10. Tr1 cells may have an influence on the Th1/Th2 balance by increasing the development of Th2 cells and decreasing the activity of Th1 cells [24], [25], [26]. This activity of SEB may thus contribute to a persistent Th2 cell bias.

In conclusion this study analyzed the intra-cytoplasmatic cytokine expression of T cells in sinonasal mucosa from healthy and CRS patients, expanding on cytokine protein measurements and supportinga high variability and co-expression of key cytokines. Whereas the majority of CD4+ and CD8+ cells expressed IFNγ intracytoplasmatically, IFNγ is hardly released without stimulation. Further, Th2 cells within the mucosal tissue, were only observed in nasal polyp patients and were increased in patients suffering from asthma and/or are positive for *Staphylococcus aureus* enterotoxin-IgE. It will be important to further characterize these obviously active and cytokine secreting Th2 cells and understand their influence in the immunological process. This knowledge may help in the development of newtherapeutic options [27], [28] which wouldreduce the number of Th2 cells, or redirect these Th2 cells into a Th1 phenotype. Tr1 regulatory cells activated by SEB may play a role in the persistence of the mucosal Th2 bias.

## Supporting Information

File S1 (DOCX)
