# Supplementary material for: Mixed T Helper Cell Signatures In Chronic Rhinosinusitis with and without Polyps
Source: PLoS One. 2014 Jun 9;9(6):e97581. doi: 10.1371/journal.pone.0097581 (PMC4049589; doi:10.1371/journal.pone.0097581)

**Online repository: Mixed T cell signatures in chronic rhinosinusitis with and without polyps**

**Material and Methods**

**Patients**

Patients were recruited at the department of Otorhinolaryngology of the Ghent University Hospital, Belgium. Inferior turbinate samples from patients without sinus disease undergoing septoplasty or rhinoseptoplasty were collected as controls (controls n=7). Samples from patients suffering from chronic rhinosinusitis (CRSsNP n=8, CRSwNP n=15 and cystic fibrosis (CF)-NP n=5) were obtained during functional endoscopic sinus surgery procedures. The diagnosis of sinus disease was based on history, clinical examination, nasal endoscopy and computed tomography of the paranasal cavities according to the current European EPOS guidelines [12]. All patients underwent a skin prick test to common inhalant allergens and terminated oral and nasal corticoids at least 4 weeks before operation. Diagnosis of asthma was obtained from the department of pneumology at the Ghent University Hospital following the GINA guidelines. 6 out of 14 patients in the CRSwNP group were asthmatic, but none in the control or CRSsNP groups (table 1). The study was approved by the local Ethical committee of the University Hospital Ghent, Belgium (B67020072535). A written informed consent was obtained from each patient before inclusion in the study.

**Characterization of T cell subtypes**

Cells were harvested, centrifuged, washed and ready for intracellular cytokine staining (ICCS) to characterize and define the different T cell subsets. Tissue single cells were first stained with LIVE/DEAD® Fixable Near-IR dye (Invitrogen) (1µl/10^6^ cells). After washing, cell pellet was dissolved in FACS buffer (1X PBS, 2% FBS, 0.05% Na-azide) and stained with different antibodies against cell surface molecules (table x) for 15 min at room temperature and followed by a washing step. After fixation and permeabilization, cells were stained with antibodies against different cytokines (Table S1). The cells were analyzed by BD FACS Canto II using FACS Diva software **(**BD Bioscience**).** Different T subsets were defined as follow: all Th subsets were CD3+CD4+CD45RA- and IFNγ + for Th1 cells, IL-4 and/or IL-5 for Th2 cells, IL-17 for Th17 cells, IL-22 for Th22 cells, IL-17 and IFNγ and IL-22 and IL-21 for Th1/17 cells, IL-21 for Tfh cells or IL-10 for Tr1 cells. Tc cells were defined as CD3+CD8+CD45RA- and IFNγ for Tc1 cells or IL-17 for Tc17 cells.

**Statistics**

The data generated in this study were analyzed using the SPSS software version 19.

The Mann-Whitney *U*–test was applied to evaluate the statistical difference between two patient groups. Data comparison within different patient groups was performed using the Kruskal-Wallis test. Correlations were calculated using the Spearman correlation coefficient. *P* values of 0.05 were regarded as significant

Table E1: Antibodies used for flow cytometry

| Antigen | Clone | Fluorochrome | Company |
| --- | --- | --- | --- |
| CD3 | SK7 | PECY7 | BD bioscience |
| CD4 | RPA-T4 | PERCPCY5.5 | ebioscience |
| CD8 | RPA-T8 | V500 | BD bioscience |
| IL-4 | MP4-25D2P | APC | BD |
| IL-5 | TRFK5 | PE | ebioscience |
| IL-10 | JES3-9D7 | APC/V450 | ebioscience |
| IL-17 | Ebio64DEC17 | AF647 | ebioscience |
| IL-21 | 3A3-N2 | PE | ebioscience |
| IL-22 | 22URTI | PE | ebioscience |
| IFNγ | 4SB3 | FITC | ebioscience |

Figure Legends

**Figure S1: Induced cytokine release in control and disease nasal mucosa**

The data are presented in scatter plots, showing the total CD3+CD4+T cells in unstimulated condition (a), nasal mucosa stimulated with PMA/Ionomycin (b).


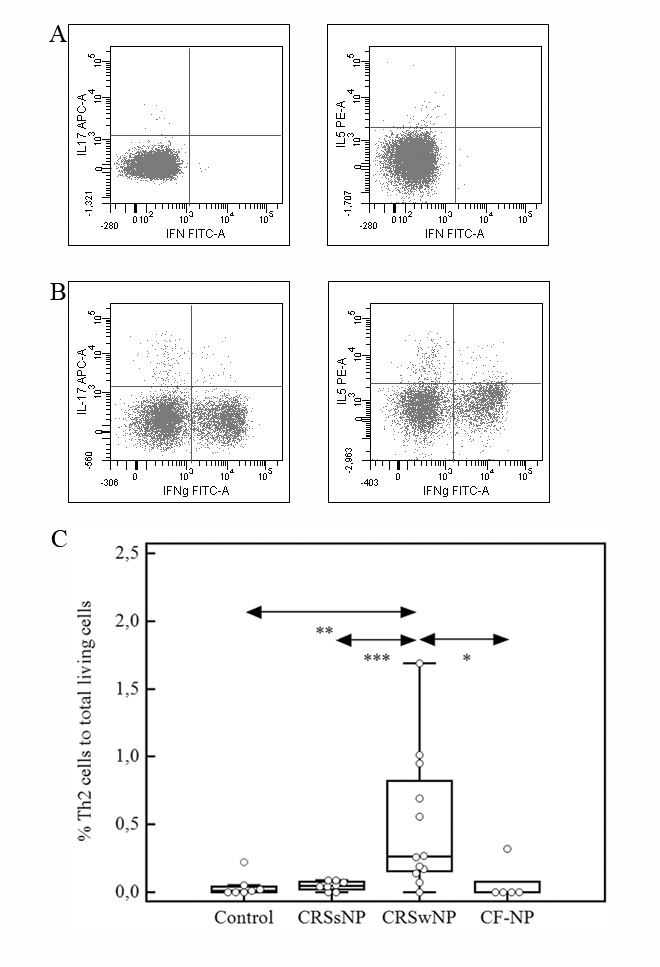

Supplement: File S1 — (DOCX) [file pone.0097581.s001.docx]
